# Use of tocilizumab and sarilumab alone or in combination with corticosteroids for covid-19: systematic review and network meta-analysis

**DOI:** 10.1136/bmjmed-2021-000036

**Published:** 2022-02-28

**Authors:** Dena Zeraatkar, Ellen Cusano, Juan Pablo Díaz Martínez, Anila Qasim, Sophia Mangala, Elena Kum, Jessica Julia Bartoszko, Tahira Devji, Thomas Agoritsas, Gordon Guyatt, Ariel Izcovich, Assem M Khamis, Francois Lamontagne, Bram Rochwerg, Per Vandvik, Romina Brignardello-Petersen, Reed Alexander Cunningham Siemieniuk

**Affiliations:** 1 Health Research Methods, Evidence, and Impact, McMaster University, Hamilton, ON, Canada; 2 Harvard Medical School, Boston, MA, USA; 3 Internal Medicine Residency Programme, University of Calgary Cumming School of Medicine, Calgary, AB, Canada; 4 Institute of Health Policy, Management and valuation, University of Toronto, Toronto, ON, Canada; 5 McMaster University, Hamilton, ON, Canada; 6 Division of General Internal Medicine & Division of Epidemiology, University Hospitals of Geneva, Geneva, Switzerland; 7 Internal Medicine, Hospital Alemán de Buenos Aires, Buenos Aires, Argentina; 8 Wolfson Palliative Care Research Centre, Hull York Medical School, Hull, UK; 9 University of Sherbrooke, Sherbrooke, QC, Canada; 10 Department of Medicine, Innlandet Hospital Trust-divisjon Gjøvik, Gjøvik, Norway

**Keywords:** COVID-19, critical care

## Abstract

**Objective:**

To compare the effects of interleukin 6 receptor blockers, tocilizumab and sarilumab, with or without corticosteroids, on mortality in patients with covid-19.

**Design:**

Systematic review and network meta-analysis.

**Data sources:**

World Health Organization covid-19 database, a comprehensive multilingual source of global covid-19 literature, and two prospective meta-analyses (up to 9 June 2021).

**Review methods:**

Trials in which people with suspected, probable, or confirmed covid-19 were randomised to interleukin 6 receptor blockers (with or without corticosteroids), corticosteroids, placebo, or standard care. The analysis used a bayesian framework and assessed the certainty of evidence using the GRADE approach. Results from the fixed effect meta-analysis were used for the primary analysis.

**Results:**

Of 45 eligible trials (20 650 patients) identified, 36 (19 350 patients) could be included in the network meta-analysis. Of 36 trials, 27 were at high risk of bias, primarily due to lack of blinding. Tocilizumab, in combination with corticosteroids, suggested a reduction in the risk of death compared with corticosteroids alone (odds ratio 0.79, 95% credible interval 0.70 to 0.88; 35 fewer deaths per 1000 people, 95% credible interval 52 fewer to 18 fewer per 1000; moderate certainty of evidence), as did sarilumab in combination with corticosteroids, compared with corticosteroids alone (0.73, 0.58 to 0.92; 43 fewer per 1000, 73 fewer to 12 fewer; low certainty). Tocilizumab and sarilumab, each in combination with corticosteroids, appeared to have similar effects on mortality when compared with each other (1.07, 0.86 to 1.34; eight more per 1000, 20 fewer to 35 more; low certainty). The effects of tocilizumab (1.12, 0.91 to 1.38; 20 more per 1000, 16 fewer to 59 more; low certainty) and sarilumab (1.07, 0.81 to 1.40; 11 more per 1000, 38 fewer to 55 more; low certainty), when used alone, suggested an increase in the risk of death.

**Conclusion:**

These findings suggest that in patients with severe or critical covid-19, tocilizumab, in combination with corticosteroids, probably reduces mortality, and that sarilumab, in combination with corticosteroids, might also reduce mortality. Tocilizumab and sarilumab, in combination with corticosteroids, could have similar effectiveness. Tocilizumab and sarilumab, when used alone, might not be beneficial.

**What is already known on this topic**

- Interleukin 6 receptor blockers have immunomodulatory effects that might be important in patients with covid-19 with immune system dysfunction and inflammation
- Corticosteroids probably reduce the risk of death in patients with severe or critical covid-19

**What this study adds**

- This systematic review and network meta-analysis provides a comprehensive review of the evidence looking at the effects of interleukin 6 receptor blockers, alone or when used in combination with corticosteroids, in covid-19
- In patients with severe or critical covid-19, tocilizumab, in combination with corticosteroids, probably reduces mortality; sarilumab, in combination with corticosteroids, could reduce mortality. Tocilizumab and sarilumab, when used without corticosteroids, might not be beneficial
- Tocilizumab and sarilumab in combination with corticosteroids could have similar effectiveness at reducing mortality

**How this study might affect research, practice, or policy**

- This review informed WHO guidelines on interleukin 6 receptor blockers

## Introduction

As of October 2021, there have been more than 240 million cumulative cases of covid-19 worldwide and nearly five million deaths.^1^ In an attempt to improve outcomes for patients with covid-19, investigators have, with varying results, repurposed several drugs.^2^ There is compelling evidence that corticosteroids reduce mortality in patients with severe and critical disease.^2^

Interleukin 6 receptor blockers have immunomodulatory effects that might be important in patients who have covid-19 with immune system dysfunction and inflammation, and these receptor blockers might therefore also result in a mortality benefit.^3–5^ The RECOVERY trial reported that tocilizumab reduces mortality and the need for invasive mechanical ventilation, particularly among patients receiving corticosteroids,^6^ and the REMAP-CAP trial reported reduced mortality and improved organ support-free days with tocilizumab and sarilumab.^7^ Although results from other trials have not been consistent,^8–10^ a prospective pairwise meta-analysis also reported that tocilizumab reduces mortality.^11^

Whether sarilumab reduces mortality and its effect relative to tocilizumab remains uncertain. Tocilizumab is not available in all settings, and because of its expense, clinicians often give the drug to only a minority of patients who might benefit.^12^ If sarilumab’s effects are comparable to those of tocilizumab, it might increase availability for patients with covid-19 who would not have otherwise have access to an interleukin 6 receptor blocker.

Further, corticosteroids are now recommended for patients with severe or critical covid-19 and, like corticosteroids, interleukin 6 receptor blockers target inflammation.^13^ Whether these receptor blockers offer any incremental benefits above corticosteroids is unknown.^13^ A prospective, pairwise meta-analysis reported that tocilizumab reduces mortality when used alone or with corticosteroids but with greater effects when combined with corticosteroids.^11^

To inform recommendations for the World Health Organization living guidelines on drugs for covid-19 treatment, we conducted a systematic review and network meta-analysis to look at the effectiveness of interleukin 6 receptor blockers, alone or in combination with corticosteroids, for patients with covid-19.^13^ This review capitalises on the methods and data of our living systematic review and network meta-analysis of drug treatments for covid-19 and represents a comprehensive and rigorous assessment of the evidence on these receptor blockers.^2^

**Figure 1:**
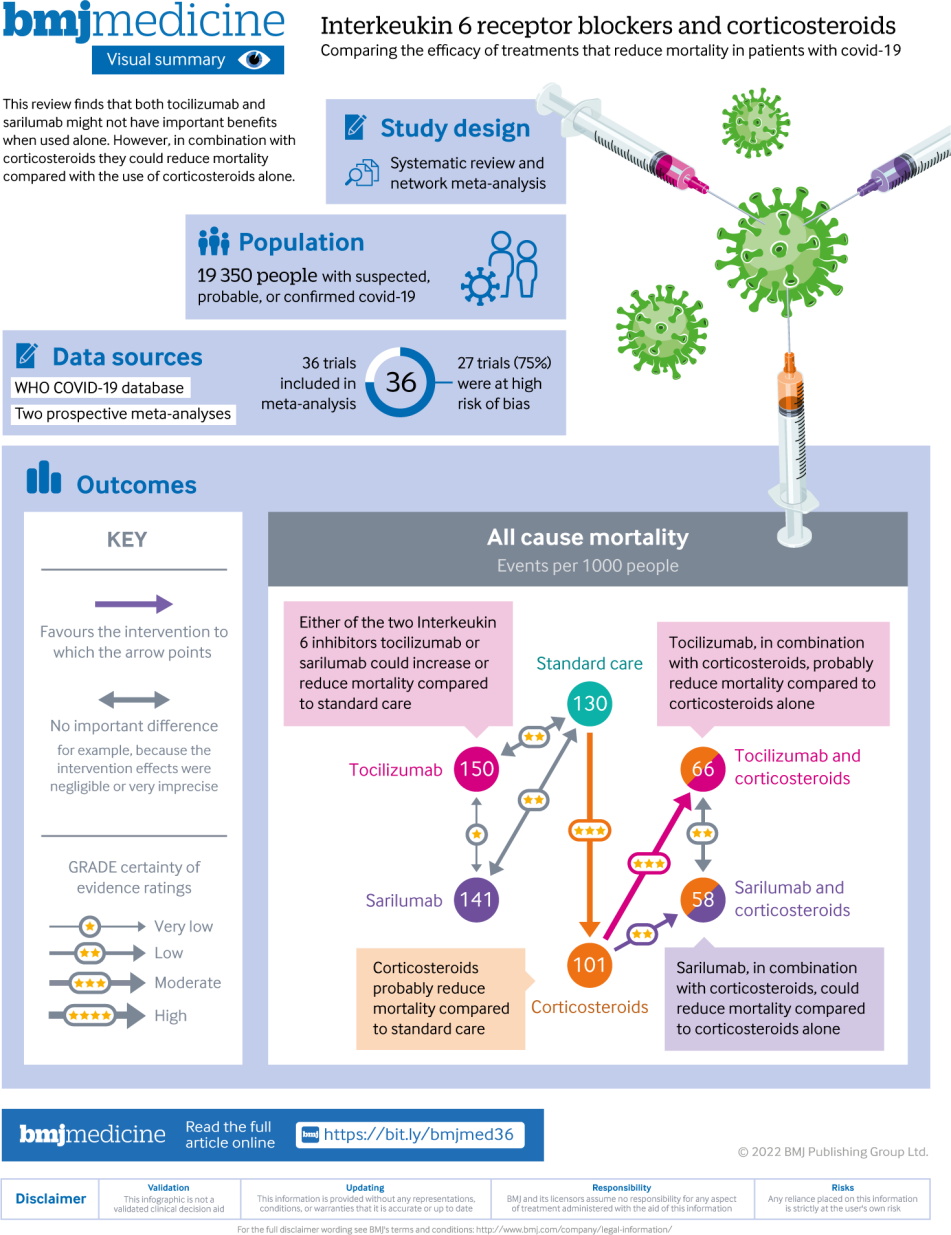
Visual summary

This systematic review and network meta-analysis is distinct from our living review of drug treatment in two ways. Firstly, in this review, we consider tocilizumab and sarilumab separately to assess their comparative effectiveness whereas our living review combines classes of the same drug within the same node. Secondly, in this review, we separate tocilizumab and sarilumab, based on concomitant use of corticosteroids, into different nodes to assess possible interactions with corticosteroids. For the visual summary of this paper, see figure 1.

## Methods

A supplement to our living systematic review and network meta-analysis of drug treatments for covid-19 includes a protocol of our methods.^2^

### Search

The present study uses the search strategy of our living review.^2^ A supplement to our drug treatment publication includes the full strategy.^2^ Briefly, we performed daily searches of the WHO covid-19 database—a comprehensive multilingual source of global published and preprint literature on covid-19 (https://search.bvsalud.org/global-literature-on-novel-coronavirus-2019-ncov/). Prior to its merging with the WHO covid-19 database on 9 October 2020, we searched the US Centres for Disease Control and Prevention's covid-19 research articles downloadable database. Our search also included six Chinese databases: Wanfang, Chinese Biomedical Literature, China National Knowledge Infrastructure, VIP, Chinese Medical Journal Net (preprints), and ChinaXiv (preprints). A validated machine learning model facilitated efficient identification of randomised trials.^14^ We searched WHO information sources from 1 December 2019 to 9 June 2021 and the Chinese literature from conception of the databases to 20 February 2021.

Our team supplemented the search by ongoing surveillance of the Living Overview of the Evidence covid-19 platform by the Epistemonikos Foundation (https://app.iloveevidence.com/loves/5e6fdb9669c00e4ac072701d) and the Norwegian Institute of Public Health's systematic and living map on covid-19 evidence (https://www.fhi.no/en/qk/systematic-reviews-hta/map/). We also included data from two WHO-sponsored prospective meta-analyses.^11 15^

### Study selection

As part of the living systematic review and network meta-analysis,^2^ pairs of reviewers, following calibration exercises, worked independently and in duplicate to screen titles and abstracts of search records and subsequently the full texts of records determined potentially eligible at the title and abstract screening stage. We linked preprint reports with their subsequent publications based on trial registration numbers, authors, and other trial characteristics. Reviewers resolved discrepancies by discussion, and when necessary, by adjudication with a third party reviewer.

This review included preprint and peer reviewed reports of trials that compared interleukin 6 receptor blockers with standard care, placebo, or corticosteroids or that compared corticosteroids with standard care or placebo in patients with suspected, probable, or confirmed covid-19. We did not set any restrictions on severity of illness, setting, or language of publication.

### Data collection

As part of the living systematic review and network meta-analysis,^2^ for each eligible trial, pairs of reviewers, following training and calibration exercises, independently extracted trial characteristics (trial registration, publication status, study design), patient characteristics (country, age, sex, type of care, severity of covid-19 symptoms), and outcomes of interest (number of participants analysed and number of participants who experienced an event) using a standardised, pilot tested data extraction form. Reviewers resolved discrepancies by discussion and, when necessary, with adjudication by a third party. We updated our data when a study preprint became available as a peer reviewed publication. For this review, we focused on all cause mortality closest to 90 days.

To assess risk of bias, reviewers, following training and calibration exercises, used a revision of the Cochrane tool for assessing risk of bias in randomised trials (RoB 2.0).^16^ Reviewers resolved discrepancies by discussion and, when necessary, by third party adjudication. A supplement to our drug treatment publication includes our modified risk-of-bias tool.^2^

### Statistical analysis

Our network meta-analysis compared tocilizumab with corticosteroids, tocilizumab without corticosteroids, sarilumab with corticosteroids, sarilumab without corticosteroids, corticosteroids, and standard care or placebo, using a bayesian framework with a plausible prior for the variance parameter and a uniform prior for the effect parameter.^17^ We summarised the effect of interventions on mortality using odds ratios and corresponding 95% credible intervals.

We classified trials in which all patients randomised to tocilizumab or sarilumab received or did not receive corticosteroids into (1) tocilizumab or sarilumab nodes with corticosteroids or (2) tocilizumab or sarilumab nodes without corticosteroids, respectively. For trials in which some patients received corticosteroids in combination with tocilizumab or sarilumab, we used subgroup data within trials to split trial participants into tocilizumab or sarilumab nodes with corticosteroids and tocilizumab or sarilumab nodes without corticosteroids. The same approach was used for standard care. We grouped patients in the standard care arm who received corticosteroids into the corticosteroid node and patients in the standard care arm who did not receive corticosteroids into the standard care without corticosteroids node. We classified trials that compared corticosteroids with standard care or placebo into corticosteroids and standard care nodes.

We performed network meta-analysis using the gemtc package of R version 3.6.3 (RStudio, Boston, MA) and pairwise meta-analyses using the bayesmeta package. Three Markov chains with 100 000 iterations after an initial burn-in of 10 000 and a thinning of 10 and used node splitting models were used to assess local incoherence and to obtain indirect estimates. We produced network plots using the network map command of Stata version 17.0 (StataCorp, College Station, TX).^18^

We performed both fixed effect and random effects network meta-analysis. Because estimates from the random effects model proved to have credible intervals that were implausibly wide owing to the uncertainty around the heterogeneity estimate, we presented results from the fixed effect meta-analysis as the primary analysis and random effects meta-analysis as a sensitivity analysis.^19^

### Certainty of evidence

To facilitate interpretation of results, we calculated absolute effects for mortality using baseline risk data from the Centres for Disease Control and Prevention on patients who were admitted to hospital for covid-19.^20 21^ We assessed the certainty of evidence using a minimally contextualised GRADE approach (grading of recommendations, assessment, development, and evaluations) for network meta-analysis with a null effect as the threshold of importance.^22–25^ The minimally contextualised approach considers only whether credible intervals include the null effect and does not consider whether plausible effects, captured by credible intervals, include both important and trivial effects. Based on a survey of the authors of our living systematic review and network meta-analysis, to evaluate certainty of no benefit (or no effect), we used a 1% risk difference threshold of the 95% credible interval.

Two reviewers with experience in applying the GRADE approach rated each domain for each comparison and resolved discrepancies by consensus. Reviewers rated the certainty for each comparison and outcome as high, moderate, low, or very low, based on considerations of risk of bias, inconsistency, indirectness, publication bias, intransitivity, incoherence (difference between direct and indirect effects), and imprecision.

### Patient and public involvement

Patients were involved in outcome selection, interpretation of results, and the generation of parallel recommendations, as part of the WHO Rapid Recommendations initiative, in partnership with *The BMJ* and MAGIC Evidence Ecosystem Foundation.^13^ Our results will be disseminated according to WHO recommendations.

## Results

### Study characteristics

**Figure 2:**
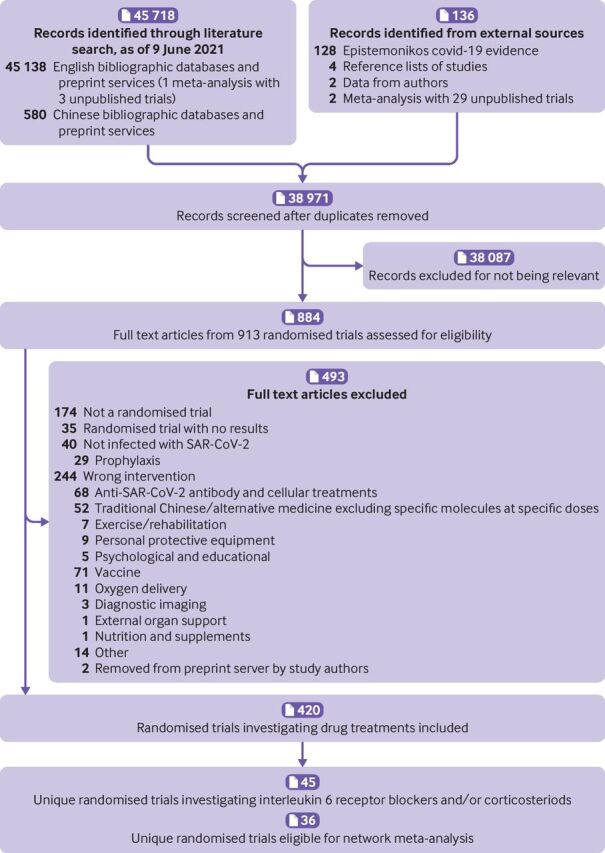
PRISMA diagram of selection of trials comparing tocilizumab and sarilumab for covid-19, alone or in combination with corticosteroids, and trials comparing corticosteroids with standard care or interleukin 6 receptor blockers

Of 45 854 titles and abstracts and 884 full texts screened, 45 trials including 20 650 patients^6 26–48^ were eligible. figure 2 presents details regarding study selection. All publications were in English. Twenty one of these trials were published, four were available as preprints, and 20 were unpublished and retrieved from two prospective meta-analyses.^11 15^

Table 1 presents trial characteristics. Twenty trials (7608 patients) compared tocilizumab with standard care or placebo^6 26–34^; seven (2756 patients) compared sarilumab with standard care or placebo with or without corticosteroids^35 36^; one (1818 patients) compared tocilizumab, sarilumab, and standard care^7^; three (366 patients) compared interleukin 6 receptor blockers with corticosteroids^48^; and 14 (8102 patients) compared corticosteroids with standard care or placebo.^37–47^

**Table 1:** Characteristics of trials comparing tocilizumab and sarilumab for covid-19, alone or in combination with corticosteroids, and trials comparing corticosteroids with standard care or interleukin 6 receptor blockers

Study	Publication status (registration)	No of participants	Country	Mean age (years)	Male (%)	Type of care (%)	Severity (based on WHO classification; %)^50^	Detailed ventilation (%)	Treatments
**Interleukin 6 receptor blocker—tocilizumab**									
ARCHITECTS	Data from meta-analysis (NCT04412772)	21	US	61.5	57.1	Inpatient	Mild/moderate (0); severe (100)	Not reported	Tocilizumab, placebo
CORIMUNO-TOCI-ICU	Data from meta-analysis (NCT04331808)	92	France	64.2	71.7	Inpatient intensive care (100.0)	Mild/moderate (0); severe (100)	High flow, non-invasive ventilation, or invasive ventilation (100.0)	Tocilizumab, standard care
COV-AID	Data from meta-analysis (NCT04330638)	230	Belgium	63.6	77.4	Inpatient	Mild/moderate (0); severe (100)	Not reported	Tocilizumab, siltuximab, standard care
COVIDOSE2-SSA	Data from meta-analysis (NCT04479358)	28	US	65	67.9	Inpatient	Mild/moderate (0); severe (100)	Mechanical ventilation (0.0%)	Tocilizumab, standard care
COVIDSTORM	Data from meta-analysis (NCT04577534)	39	Finland	65.7	53.9	Inpatient	Mild/moderate (0); severe (100)	Not reported	Tocilizumab, standard care
COVITOZ	Data from meta-analysis (NCT04435717)	26	Spain	57.5	65.4	Inpatient, intensive care (0.0)	Mild/moderate (100); severe (0)	Not reported	Tocilizumab, standard care
Hermine, 2021; CORIMUNO-TOCI 1^26^	Published (NCT04331808)	131	France	64	67.7	Inpatient, intensive care (0.0)	Mild/moderate (0); severe (100); critical (0)	Supplemental oxygen (100.0); non-invasive ventilation or invasive ventilation (0.0)	Tocilizumab, standard care
HMO-020–0224	Data from meta-analysis (NCT04377750)	54	Israel	63.1	68.5	Inpatient	Mild/moderate (0); severe (100)	Not reported	Tocilizumab, placebo
Horby, 2021; RECOVERY^6^	Published (NCT04381936)	4116	UK	63.6	67.3	Inpatient	Mild/moderate (0); severe (100)	Supplemental oxygen (45.4); high flow or non-invasive ventilation (41.0); invasive ventilation(13.7)	Tocilizumab, standard care
ImmCoVA	Data from meta-analysis (NCT04412291)	49	Sweden	Not reported	Not reported	Inpatient	Mild/moderate (0); severe (100)	invasive ventilation (0.0)	Tocilizumab, standard care
REMDACTA	Data from meta-analysis (NCT04409262)	640	Spain, US, Brazil, Russia	60.3	63.3	Inpatient	Mild/moderate (0); severe (100)	Not reported	Tocilizumab, placebo
Rosas, 2021; COVACTA^27^	Published (NCT04320615)	452	Canada, Denmark, France, Germany, Italy, Netherlands, Spain, UK, US	60.8	69.9	Inpatient, intensive care (56.4)	Mild/moderate (0); severe (100); critical (0)	Non-invasive ventilation or invasive ventilation (37.7)	Tocilizumab, placebo
Rutgers, 2021; PreToVid^28^	Preprint (NL8504)	354	Netherlands	66.5	67	Inpatient, intensive care (0.0)	Mild/moderate (0); severe (100)	Supplemental oxygen (96.6)	Tocilizumab, standard care
Soin, 2021; COVINTOC^29^	Published (CTRI/2020/05/025369)	180	India	55	84.9	Inpatient	Mild/moderate (49.2); severe (50.8)	Supplemental oxygen (89.9); non-invasive ventilation (26.8); invasive ventilation (5.0)	Tocilizumab, standard care
Stone, 2020; BACC BAY^30^	Published (NCT04356937)	243	US	59.9	58	Inpatient, intensive care (4.5)	Mild/moderate (0); severe (100); critical (0)	Supplemental oxygen (79.8); high flow or non-invasive ventilation (4.1); invasive ventilation (0.4)	Tocilizumab, placebo
Salama, 2021; EMPACTA^31^	Published (NCT04372186)	388	US, Peru, Brazil, Kenya, South Africa, Mexico	55.9	59.1	Inpatient, intensive care (15.4)	Mild/moderate (0); severe (100); critical (0)	Mechanical ventilation (0.0)	Tocilizumab, placebo
Salvarani, 2020; RCT-TCZ-COVID-19^32^	Published (NCT04346355)	126	Italy	60	61.1	Inpatient, intensive care (0.0)	Mild/moderate (0); severe (100); critical (0)	non-invasive ventilation or invasive ventilation (0.0)	Tocilizumab, standard care
TOCOVID	Data from meta-analysis (EudraCT2020-001442-19)	270	Spain	53	63.7	Inpatient, intensive care (100.0)	Mild/moderate (0); severe (100)	Not reported	tocilizumab standard care
Talaschian, 2021 33	Preprint (RCT20081027001411N4)	40	Iran	61.7	52.8	Inpatient	Mild/moderate (0); severe (100); critical (0)	Nasal cannula (50.0); simple mask (30.6); reservoir mask (11.1); non-invasive ventilation (8.3); invasive ventilation (0.0)	Tocilizumab, standard care
Veiga, 2021; TOCIBRAS^34^	Published (NCT04403685)	129	Brazil	57.5	68.2	Inpatient	Mild/moderate (0); severe (100)	Supplemental oxygen (51.9); high flow or non-invasive ventilation (31.8); invasive ventilation (16.3)	Tocilizumab, standard care
**Interleukin 6 receptor blocker—sarilumab**									
CORIMUNO-SARI-1	Data from meta-analysis (NCT04324073)	144	France	62.3	75	Inpatient, intensive care (0.0)	Mild/moderate (0); severe (100)	Not reported	Sarilumab, standard care
CORIMUNO-SARI-ICU	Data from meta-analysis (NCT04324073)	81	France	61.6	76.5	Inpatient, intensive care (100.0)	Mild/moderate (0); severe (100)	Not reported	Sarilumab, standard care
SARCOVID	Data from meta-analysis (NCT04357808)	30	Spain	61.7	66.7	Inpatient	Mild/moderate (0); severe (100)	Mechanical ventilation (0.0)	Sarilumab, standard care
SARICOR	Data from meta-analysis (EudraCT2020-001531-27)	80	Spain	59.4	71.2	Inpatient	Mild/moderate (0); severe (100)	Mechanical ventilation (0.0)	Sarilumab, standard care
SARTRE	Data from meta-analysis (EudraCT2020-002037-15)	140	Spain	58.4	72.9	Inpatient, intensive care (0.0)	Mild/moderate (0); severe (100)	Mechanical ventilation (0.0)	Sarilumab, standard care
Sivapalasingam, 2021 (phase 2); Sarilumab-COVID-19^35^	Preprint (NCT04315298)	457	US	58.7	72.4	Inpatient	Mild/moderate (0); severe (50.5); critical (49.5)	Supplemental oxygen (27.6); high flow, non-invasive ventilation, or invasive ventilation (49.5)	Sarilumab (200 mg), sarilumab (400 mg), placebo
Sivapalasingam, 2021 (phase 3, cohort 1); Sarilumab-COVID-19^35^	Preprint (NCT04315298)	1365	US	61.7	64.5	Inpatient	Mild/moderate (0); severe (45); Critical (55)	Supplemental oxygen (27.0); invsive ventilation (21.8)	Sarilumab (200 mg), sarilumab (400 mg), placebo
Sivapalasingam, 2021 (phase 3, cohort 2); Sarilumab-COVID-19^35^	Preprint (NCT04315298)	31	US	48.7	71	Inpatient	Mild/moderate (0); severe (0); critical (100)	IV (100.0)	Sarilumab (800 mg), placebo
Sivapalasingam, 2021 (phase 3, cohort 3); Sarilumab-COVID-19^35^	Preprint (NCT04315298)	8	US	60.8	62.5	Inpatient	Mild/moderate (0); severe (0); critical (100)	High flow or non-invasive ventilation (100.0); invasive ventilation (0.0)	Sarilumab (800 mg), placebo
Lescure, 2021^36^	Published (NCT04327388; Eudra CT (2020-001162-12), WHO (U1111-1249-6021))	420	Argentina, Brazil, Canada, Chile, France, Germany, Israel, Italy, Japan, Russia, Spain	59	62.7	Inpatient, intensive care (35.6)	Mild/moderate (0); severe (60.6); critical (38.9)	Nasal cannula (42.1); face mask (26.7); non-rebreather face mask (10.5); high flow (6.2); non-invasive ventilation (1.7); invasive ventilation (11.5); other (1.2)	Sarilumab (400 mg), sarilumab (200 mg), placebo
**Interleukin 6 receptor blockers—tocilizumab/sarilumab**									
Gordon, 2021; REMAP-CAP^51^	Published (NCT02735707)	798	UK, Netherlands, Australia, New Zealand, Ireland, Saudia Arabia	61.4	72.6	Inpatient, intensive care (100.0)	Mild/moderate (0); severe (0); critical (100)	High flow (28.8); non-invasive ventilation (41.5); invasive ventilation (29.4)	Tocilizumab standard care (for tociluzumab), sarilumab standard care (for sarilumab)
Gordon, 2021; REMAP-CAP^51^	Data from authors (NCT02735707)	1020	UK, Netherlands, Australia, New Zealand, Ireland, Saudia Arabia	Not reported	Not reported	Inpatient	Mild/moderate (0); severe (0); critical (100)	Not reported	Tocilizumab, sarilumab
**Interleukin 6 receptor blockers *v* corticosteroids**									
Rashad, 2021^48^	Published (CT04519385 (19/08/2020))	149	Egypt	62.5	56.9	Inpatient, intensive care (100.0)	Mild/moderate (0); severe (100); critical (100)	Non-invasive ventilation (64.2); invasive ventilation (35.8)	Tocilizumab, dexamethasone
SILCOR	Data from meta-analysis (EudraCT2020-001413-20)	158	Spain	62	65.2	Inpatient	Mild/moderate (0); severe (100); critical (0)	Not reported	Siltuximab, corticosteroids
STORM	Data from meta-analysis (NCT04345445)	59	Malaysia	53.2	76.3	Inpatient, intensive care (0.0)	Mild/moderate (100); severe (0); critical (0)	Mechanical ventilation (0.0)	Tocilizumab, dexamethasone
**Corticosteroids**									
Angus, 2020; REMAP-CAP^37^	Published (NCT02735707)	403	Australia, Canada, Ireland, France, Netherlands, New Zealand, UK, US	59.9	71.1	Inpatient, intensive care (100.0)	Mild/moderate (0); severe (100)	High flow (14.6); non-invasive ventilation (29.7); invasive ventilation (55.5); extracorporeal membrane oxygenation (0.8)	Hydrocortisone (fixed dose), hydrocortisone (shock dependent), standard care
Corral-Gudino, 2021; GLUCOCOVID^38^	Published (2020-001934-37)	64	Spain	69.8	60.9	Inpatient, intensive care (0.0)	Mild/moderate (0); severe (100); critical (0)	Mechanical ventilation (0.0)	Methylprednisolone, standard care
Dequin, 2020 CAPECOVID^39^	Published (NCT02517489)	149	France	62.2	69.8	Inpatient, intensive care (100.0)	Mild/moderate (0); severe (0); critical (100)	Non-rebreathing mask with a reservoir bag (6.0); high flow (12.8); non-invasive ventilation or invasive ventilation (81.2)	Hydrocortisone, placebo
Edalatifard, 2020^40^	Published (IRCT20200404046947N1)	68	Iran	58.5	62.9	Inpatient	Mild/moderate (0); severe (100); critical (0)	Nasal cannula (21.0); simple mask (11.3); reserve mask (29.0); non-invasive ventilation (37.1)	Methylprednisolone, standard care
Farahani, 2020^41^	Preprint (IRCT20200406046963N1)	29	Iran	64	65.5	Inpatient, intensive care (100.0)	Mild/moderate (0); severe (100)	Not reported	Methylprednisolone, prednisolone standard care
Horby, 2021 RECOVERY^42^	Published (NCT04381936)	6425	UK	66.2	63.6	Inpatient	Not reported	Supplemental oxygen or non-invasive ventilation (60.4); invasive ventilation or extracorporeal membrane oxygenation (15.7)	Dexamethasone, standard care
Jamaati, 2021^43^	Published (IRCT20151227025726N17)	50	Iran	62	72	Inpatient	Mild/moderate (0); severe (100)	Not reported	Dexamethasone, standard care
Jeronimo, 2020; Metcovid^44^	Published (NCT04343729)	416	Brazil	55	65.3	Inpatient, intensive care (35.4)	Not reported	Non-invasive oxygen (47.5); invasive mechanical ventilation (33.9)	Methylprednisolone, placebo
Steroids-SARI	Data from meta-analysis (NCT04244591)	47	China	64.5	74.5	Inpatient, intensive care (100.0)	Mild/moderate (0); severe (0); critical (100)	Mechanical ventilation (57.5)	Methylprednisolone, standard care
DEXA-COVID 19	Data from meta-analysis (NCT04325061)	19	Spain	60.7	68.4	Not reported	Mild/moderate (0); severe (0); critical (100)	Invasive ventilation (100.0)	Dexamethasone, standard care
COVID STEROID	Data from meta-analysis (NCT04348305)	29	Denmark	59.4	79.3	Not reported	Mild/moderate (0); severe (0); critical (100)	Mechanical ventilation (51.7)	Hydrocortisone, placebo
Tang, 2021^45^	Published (NCT04273321)	86	China	56	47.7	Inpatient, intensive care (0.0)	Mild/moderate (0); severe (100)	Nasal cannula (70.9)	Methylprednisolone, standard care
Tomazini, 2020; CoDEX^46^	Published (NCT04327401)	299	Brazil	61.4	62.5	Inpatient, intensive care (100.0)	Mild/moderate (0); severe (0); critical (100)	Pressure control ventilation (44.5); volume control ventilation (46.5); other (9.0)	Dexamethasone, standard care
Vaira, 2020^47^	Published	18	Italy	42.1	38.9	Outpatient, intensive care (0.0%)	Mild/moderate (100); severe (0); critical (0)	Not reported	Corticosteroids, standard care

One trial, REMAP-CAP,^7^ randomised patients to tocilizumab or standard care (among centres with access to tocilizumab) or to sarilumab or standard care (among centres with access to sarilumab). Randomisation to standard care was halted when an interim analysis showed efficacy of tocilizumab and sarilumab, after which patients were randomised to either tocilizumab or sarilumab, with both groups receiving corticosteroids. As such, we treated REMAP-CAP as three separate trials in our analyses (that is, tocilizumab *v* standard care; sarilumab *v* standard care; tocilizumab *v* sarilumab). We used 90 day mortality for the comparisons of tocilizumab and sarilumab with standard care and obtained data on in-hospital mortality from the investigators for the comparison of tocilizumab and sarilumab. The comparison between tocilizumab and sarilumab was restricted to patients who were eligible for randomisation to either drug in the later phase of the trial.

Another trial, Sarilumab-COVID-19, was conducted in two phases.^35^ In phase 1, researchers randomised patients to 400 mg sarilumab, 200 mg sarilumab, or placebo. A prespecified interim analysis of the first phase showed the benefit of 400 mg sarilumab in patients in the critical group (receiving high flow supplemental oxygen or mechanical ventilation) and potential harm of 400 mg sarilumab in patients in the severe group (receiving low flow supplemental oxygen) and the multisystem organ dysfunction group. Subsequently, enrolment into the severe and multisystem organ dysfunction groups and use of the 200 mg dose of sarilumab were discontinued. Thereafter, phase 2 was amended to restrict enrolment to patients in the critical group receiving mechanical ventilation with further randomisation to 400 mg sarilumab and placebo, and to add two new cohorts. These new cohorts included a group of patients with critical disease receiving mechanical ventilation who were randomised to 800 mg sarilumab or placebo (phase 3 modification 1); and a group of patients with critical disease not receiving mechanical ventilation, but requiring high flow oxygen or non-invasive ventilation, randomised to 800 mg sarilumab or placebo (phase 2 modification 2). The trial was thus treated as four separate trials (phase 1, phase 2 modification 0, phase 2 modification 1, phase 2 modification 2).

### Patient characteristics

Table 1 presents characteristics of included patients. Trials included a median of 129 participants (interquartile range 47-354). The mean age of patients in trials ranged between 42.1 to 69.8 years. About half of all patients were recruited from the UK. All but one trial reported on in-patients. Most patients had severe to critical disease and were receiving supplementary oxygen.

### Risk of bias

**Figure 3:**
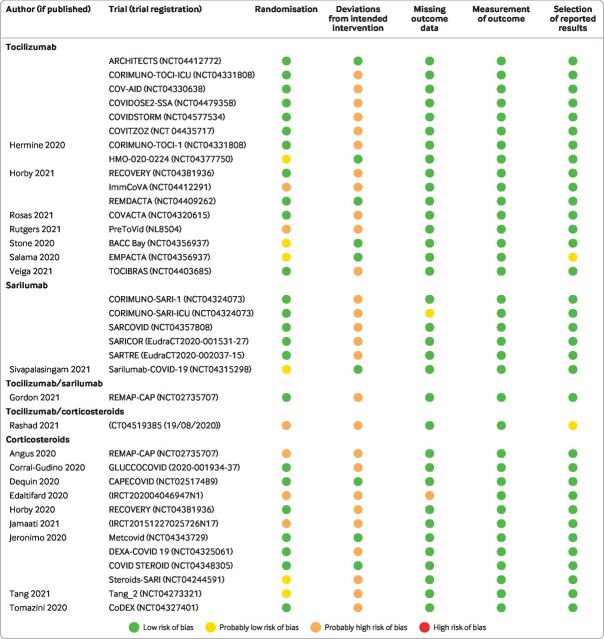
Risk of bias of trials included in network meta-analysis comparing tocilizumab and sarilumab for covid-19, alone or in combination with corticosteroids, and trials comparing corticosteroids with standard care or interleukin 6 receptor blockers

Figure 3 presents risk-of-bias assessments for the trials included in the analysis. Nine trials (including 3801 participants) were rated as low risk of bias and the remainder (27 trials; 15 549 participants) were at high risk of bias—primarily due to a lack of blinding.

### Mortality

**Figure 4:**
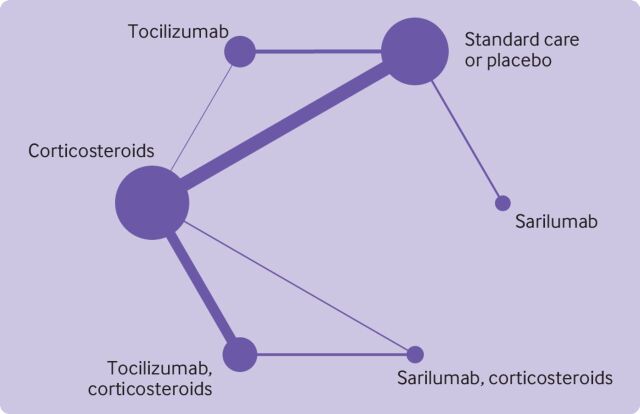
Network diagram of meta-analysis comparing use of tocilizumab and sarilumab for covid-19, alone or in combination with corticosteroids. Nodes are weighted by the number of studies for each treatment, and edges are weighted by precision (inverse variance) for each pairwise comparison

The network meta-analysis included 36 trials, with 19 350 patients and 5269 deaths comparing tocilizumab and sarilumab, with or without corticosteroids, and comparing corticosteroids with standard care or placebo.^6 26–28 30 31 34 35 37–40 42–46 48^ The analysis did not include the remaining nine trials from the initial search because they either did not report outcome data, or we could not retrieve subgroup data based on concomitant treatment with corticosteroids for trials compared interleukin 6 receptor blockers with standard care or placebo.^29 32 33 36 41 47^ figure 4 presents the network plot. Online supplemental file 1 presents data for the network meta-analysis. Table 2 presents results from the network meta-analysis.

10.1136/bmjmed-2021-000036.supp1 Supplementary data

**Table 2:** Summary of findings for network meta-analysis comparing use tocilizumab and sarilumab for covid-19, alone or in combination with corticosteroids

Comparison	Odds ratio (95% CrI)	Risk difference (95% CrI)		Certainty/quality of evidence	Summary
		Intervention 1 (No of deaths/1000 people)	Intervention 2 (No of deaths/1000 people)		
Tocilizumab *v* standard care	1.12 (0.91 to 1.38)	150 per 1000	130 per 1000	Low (risk of bias, imprecision)	Tocilizumab might increase or reduce mortality compared with standard care
		Difference: 19.73 more per 1000 (−15.78 to 58.52)			
Sarilumab *v* standard care	1.07 (0.81 to 1.40)	141 per 1000	130 per 1000	Low (risk of bias, imprecision)	Sarilumab might increase or reduce mortality compared with standard care.
		Difference: 10.60 more per 1000 (−38.37 to 55.17)			
Corticosteroids *v* standard care	0.84 (0.75 to 0.93)	101 per 1000	130 per 1000	Moderate (risk of bias)	Corticosteroids probably reduce mortality compared with standard care
		Difference: 29.27 fewer per 1000 (−46.74 to −12.24)			
Sarilumab *v* tocilizumab	0.95 (0.68 to 1.35)	141 per 1000	150 per 1000	Very low (risk of bias, imprecision)	The effects of sarilumab compared with tocilizumab are uncertain
		Difference: 9.13 fewer per 1000 (−74.66 to 49.13)			
Tocilizumab and corticosteroids *v* corticosteroids alone	0.79 (0.70 to 0.88)	66 per 1000	101 per 1000	Moderate (risk of bias)	Tocilizumab, in combination with corticosteroids, probably reduces mortality compared with corticosteroids alone
		Difference: 34.54 fewer per 1000 (−51.80 to −18.23)			
Sarilumab and corticosteroids *v* corticosteroids alone	0.73 (0.58 to 0.92)	58 per 1000	101 per 1000	Low (risk of bias, imprecision)	Sarilumab, in combination with corticosteroids, might reduce mortality compared with corticosteroids alone
		Difference: 42.73 fewer per 1000 (−72.61 to −12.00)			
Tocilizumab and corticosteroids *v* sarilumab and corticosteroids	1.07 (0.86 to 1.34)	66 per 1000	58 per 1000	Low (risk of bias, imprecision)	Tocilizumab, in combination with corticosteroids, could have similar effects to reduce mortality compared with sarilumab in combination with corticosteroids
		Difference: 8.19 more per 1000 (−20.49 to 34.96)			

Compared with corticosteroids alone, tocilizumab, in combination with corticosteroids, probably reduce mortality and sarilumab, in combination with corticosteroids, might reduce mortality (odds ratio 0.79, 95% credible interval 0.70 to 0.88; 34.54 fewer deaths per 1000 people, 51.80 to −18.23; moderate certainty). In combination with corticosteroids, tocilizumab could have similar effects to sarilumab in reducing mortality (1.07, 0.86 to 1.34; 8.19 more per 1000,–20.49 to 34.96; low certainty). The effects of tocilizumab and sarilumab, when used alone, are unclear and might increase or reduce mortality compared with standard care (tocilizumab 1.12, 0.91 to 1.38; 19.73 more per 1000, –15.78 to 58.52; low certainty; sarilumab 1.07, 0.81 to 1.40; 10.60 more per 1000, –38.37 to 55.17; low certainty). Online supplemental file 2 presents all direct and indirect comparisons and their certainty of evidence.

10.1136/bmjmed-2021-000036.supp2 Supplementary data

Online supplemental file 3 presents results from the random effects model, which were consistent with results from the fixed effects model—however, the random effects model produced effect estimates that were more imprecise owing to the incorporation of an additional heterogeneity parameter in the model.

10.1136/bmjmed-2021-000036.supp3 Supplementary data

## Discussion

### Principal findings

This systematic review and network meta-analysis, which includes data from 45 randomised trials and 20 650 patients (36 trials with 19 350 patients eligible for network meta-analysis), provides a comprehensive overview of the evidence for interleukin 6 receptor blockers, alone and in combination with corticosteroids. Our results show that, in patients with severe or critical covid-19, tociluzumab probably reduces mortality when added to a standard care regimen that includes corticosteroids; and sarilumab could reduce mortality when added to a standard care regimen that includes corticosteroids. We also show that sarilumab could have similar effectiveness to tocilizumab but whether interleukin 6 receptor blockers have any impact on mortality without concomitant use of corticosteroids remains uncertain.

### Comparison with other studies

Our findings are consistent with those from a prospective pairwise meta-analysis^11^ and the largest trials on interleukin 6 receptor blockers, RECOVERY and REMAP-CAP.^6 7^ While RECOVERY and REMAP-CAP reported tocilizumab and sarilumab to be effective, the observed effect could be attributed to over 80% of patients in these trials also receiving corticosteroids concomitantly.^6 7^ A subgroup analysis of RECOVERY based on baseline corticosteroids showed a reduction in mortality in the subgroup of patients who received corticosteroids at baseline, but this reduction in mortality was not observed in patients that did not receive corticosteroids.^6^ Although several smaller trials did not find evidence of a benefit with tocilizumab, this is probably because smaller individual trials were underpowered to detect such a modest reduction in mortality.

Our study adds to the evidence base by showing that interleukin 6 receptor blockers probably reduce mortality when added to a standard care regimen that includes corticosteroids, and that sarilumab could have a similar effect on mortality as tocilizumab. This result is largely driven by the REMAP-CAP trial that directly compared sarilumab to tocilizumab.

### Strengths and limitations of this study

The strengths of this review include the comprehensive search and screening strategy. In addition to trials that we identified as part of our own search, we also added trials from two pairwise, prospective meta-analyses that included an inception cohort of registered trials, thereby minimising the effects of publication bias.^11 15^

Our findings were limited by the risk of bias of the trials, most of which were at high risk of bias owing to a lack of blinding, which might have introduced bias through differences in co-interventions between randomised groups. We took a conservative approach and rated down the certainty of evidence for risk of bias, owing to possible differences in co-interventions. Some, including the linked WHO guideline panel, did not consider lack of blinding to be a serious concern for mortality because it is an objective outcome.^13^

In this review, we only considered corticosteroid use at the time of randomisation. Some patients probably received corticosteroids after randomisation, but were considered not to have received concomitant corticosteroids. Administration of corticosteroids to patients was not at random.

In the included trials, patients were not randomised to receive interleukin 6 receptor blockers alone or in combination with corticosteroids, and the comparison of interleukin 6 receptor blockers with and without corticosteroids was based on subgroup data. Corticosteroids are recommended for patients with severe or critical disease receiving supplemental oxygen or ventilation and are not recommended for patients with mild or moderate disease, so we would expect patients receiving corticosteroids to have worse outcomes than patients not receiving corticosteroids.^13^ The opposite effect was, however, observed in our study, with patients receiving interleukin 6 receptor blockers in combination with corticosteroids faring better than those receiving interleukin 6 receptor blockers alone, which further supports an interaction between interleukin 6 receptor blockers and corticosteroids.

Claims of subgroup effects or interactions are often spurious. To avoid any spurious claims, the parallel WHO guideline panel assessed the credibility of the subgroup effect using the ICEMAN tool and found it to be of high credibility.^49^ While corticosteroids are associated with respiratory support, a parallel, pairwise systematic review and meta-analysis did not find evidence of a subgroup effect based on baseline respiratory use.^11^

Four trials included in our systematic review were only available as preprint publications. Including preprints in meta-analyses could increase the precision of estimates, allow timely dissemination, and minimise the effects of publication bias. Preprints could, however, reduce the credibility of evidence syntheses and risk serious errors if important differences appear in later published reports. As part of our living systematic review and network meta-analysis, we have been maintaining a comprehensive comparison of differences in key methods and results between preprints and publications. Such differences have mostly been limited to baseline patient characteristics and any changes we have observed have not resulted in an important change to the pooled effect estimates or certainty of evidence.^2^

### Conclusion

Evidence from this systematic review and network meta-analysis indicates that in patients with severe or critical covid-19, interleukin 6 receptor blockers, when administered with corticosteroids, probably reduce mortality. The available evidence suggests that tocilizumab and sarilumab could be similarly effective. Our findings support linked WHO guidelines on interleukin 6 receptor blockers, which provides a strong recommendation for using either tocilizumab or sarilumab in combination with corticosteroids for patients with severe or critical covid-19.^13^

## Data Availability

All data relevant to the study are included in the article or uploaded as supplementary information.
