# Supplementary material for: Use of tocilizumab and sarilumab alone or in combination with corticosteroids for covid-19: systematic review and network meta-analysis
Source: BMJ Med. 2022 Feb 28;1(1):e000036. doi: 10.1136/bmjmed-2021-000036 (PMC9978750; doi:10.1136/bmjmed-2021-000036)
Supplement: Supplementary data [file bmjmed-2021-000036supp001.pdf]

Ref ID	1st Author	Intervention name	Subgroup	Dichotomous outcomes				Comments
				Outcome<br>1=Mortality [closest to 90 days]<br>2=Mechanical ventilation (includes both invasive and non-invasive) [closest to 90 days]<br>3=Admission to hospital [within 28 days]<br>4=Adverse effects leading to discontinuation [within 28 days]<br>5=Viral clearance [closest to 7 days +/- 3 days]<br>6=Venous thromboembolism [only for anticoagulants]<br>7=Clinically important bleeding [only for	Follow-up time (days)	N analyzed	Number of events	
5743, 2967	Corral-Gudino	thylprednisol	NA	1	28	35	7	NA
5743, 2967	Corral-Gudino	standard care	NA	1	28	29	5	NA
9, 7002, 33	Horby_1	examethason	NA	1	28	2104	482	NA
9, 7002, 33	Horby_1	standard care	NA	1	28	4321	1110	NA
12062	Dequin	ydrocortison	NA	1	21	75	11	NA
12062	Dequin	placebo	NA	1	21	73	20	NA
12141	Tomazini	examethason	NA	1	28	151	85	NA
12141	Tomazini	standard care	NA	1	28	148	91	NA
12146	Angus	ortisone (fixe	NA	1	NR	137	41	NA
12146	Angus	sone (shock-c	NA	1	NR	141	37	NA
12146	Angus	standard care	NA	1	NR	101	33	NA
r Patients \	Sterne	examethason	NA	1	28	7	2	NA
r Patients \	Sterne	standard care	NA	1	28	12	2	NA
D-19 and S	Sterne	hydrocortis one	NA	1	28	15	6	NA
D-19 and S	Sterne	placebo	NA	1	28	14	2	NA
Ill Patients	Sterne	thylprednisol	NA	1	30	24	13	NA
Ill Patients	Sterne	standard care	NA	1	30	23	13	NA
13208	Edalatifard	thylprednisol	NA	1	NR	34	2	NA
13208	Edalatifard	standard care	NA	1	NR	28	12	NA
2	Farahani	dnisolone, pr	NA	1	NR	14	0	NA
2	Farahani	standard care	NA	1	NR	15	NR	NA
27640	Tang_2	thylprednisol	NA	1	NR	43	0	NA
27640	Tang_2	standard care	NA	1	NR	43	1	NA
31583	Jamaati	examethason	NA	1	28	25	16	NA
31583	Jamaati	standard care	NA	1	28	25	15	NA
10699	Jeronimo	thylprednisol	NA	1	28	209	79	NA
10699	Jeronimo	placebo	NA	1	28	207	80	NA
osas, 3330	COVACTA_NCT04320615	tocilizumab	Steroids	1	90	56	16	NA
osas, 3330	COVACTA_NCT04320615	placebo	Steroids	1	90	41	14	NA
osas, 3330	COVACTA_NCT04320615	tocilizumab	No steroids	1	90	238	56	NA
osas, 3330	COVACTA_NCT04320615	placebo	No steroids	1	90	103	22	NA
30368	RECOVERY_NCT04381936	tocilizumab	Steroids	1	28	1664	482	NA
30368	RECOVERY_NCT04381936	standard care	Steroids	1	28	1721	600	NA
30368	RECOVERY_NCT04381936	tocilizumab	No steroids	1	28	357	139	NA
30368	RECOVERY_NCT04381936	standard care	No steroids	1	28	367	127	NA
ECTS_NCT0	ARCHITECTS_NCT04412772	tocilizumab	Steroids	1	90	9	0	NA
ECTS_NCT0	ARCHITECTS_NCT04412772	placebo	Steroids	1	90	11	2	NA
ECTS_NCT0	ARCHITECTS_NCT04412772	tocilizumab	No steroids	1	90	1	0	NA
ECTS_NCT0	ARCHITECTS_NCT04412772	placebo	No steroids	1	90	0	0	NA
16283	BACC Bay_NCT04356937	tocilizumab	Steroids	1	28	3	0	NA
16283	BACC Bay_NCT04356937	placebo	Steroids	1	28	1	0	NA
16283	BACC Bay_NCT04356937	tocilizumab	No steroids	1	28	158	9	NA
16283	BACC Bay_NCT04356937	placebo	No steroids	1	28	81	4	NA
16128	ORIMUNO-TOCI-1_NCT0433180	tocilizumab	Steroids	1	90	10	1	NA
16128	ORIMUNO-TOCI-1_NCT0433180	standard care	Steroids	1	90	12	4	NA
16128	ORIMUNO-TOCI-1_NCT0433180	tocilizumab	No steroids	1	90	53	6	NA
16128	ORIMUNO-TOCI-1_NCT0433180	standard care	No steroids	1	90	55	7	NA
16127	ORIMUNO-TOCI-ICU_NCT043318	tocilizumab	Steroids	1	90	8	4	NA
16127	ORIMUNO-TOCI-ICU_NCT043318	standard care	Steroids	1	90	4	2	NA
16127	ORIMUNO-TOCI-ICU_NCT043318	tocilizumab	No steroids	1	90	41	8	NA
16127	ORIMUNO-TOCI-ICU_NCT043318	standard care	No steroids	1	90	39	11	NA
ID_NCT043	COV-AID_NCT04330638	tocilizumab	Steroids	1	90	48	7	NA
ID_NCT043	COV-AID_NCT04330638	standard care	Steroids	1	90	42	3	NA
ID_NCT043	COV-AID_NCT04330638	tocilizumab	No steroids	1	90	33	3	NA
ID_NCT043	COV-AID_NCT04330638	standard care	No steroids	1	90	30	6	NA
2-SSA_NC1	COVIDOSE2-SSA_NCT04479358	tocilizumab	Steroids	1	28	6	0	NA
2-SSA_NC1	COVIDOSE2-SSA_NCT04479358	standard care	Steroids	1	28	2	1	NA
2-SSA_NC1	COVIDOSE2-SSA_NCT04479358	tocilizumab	No steroids	1	28	13	0	NA

2-SSA_NCT	COVIDOSE2-SSA_NCT04479358	standard care	No steroids	1	28	6	1	NA
6340, 2364	EMPACTA_NCT04372186	tocilizumab	Steroids	1	90	200	27	NA
6340, 2364	EMPACTA_NCT04372186	placebo	Steroids	1	90	112	15	NA
6340, 2364	EMPACTA_NCT04372186	tocilizumab	No steroids	1	90	49	2	NA
6340, 2364	EMPACTA_NCT04372186	placebo	No steroids	1	90	16	0	NA
-0224_NCT	HMO-020-0224_NCT04377750	tocilizumab	Steroids	1	90	31	16	NA
-0224_NCT	HMO-020-0224_NCT04377750	placebo	Steroids	1	90	15	9	NA
-0224_NCT	HMO-020-0224_NCT04377750	tocilizumab	No steroids	1	90	6	2	NA
-0224_NCT	HMO-020-0224_NCT04377750	placebo	No steroids	1	90	2	1	NA
1, Gordon_	AP-CAP_1_NCT02735707_tociliz	tocilizumab	Steroids	1	90	214	63	NA
1, Gordon_	AP-CAP_1_NCT02735707_tociliz	standard care	Steroids	1	90	217	80	NA
1, Gordon_	AP-CAP_1_NCT02735707_sarilu	sarilumab	Steroids	1	90	44	8	NA
1, Gordon_	AP-CAP_1_NCT02735707_sarilu	standard care	Steroids	1	90	52	13	NA
1, Gordon_	AP-CAP_1_NCT02735707_tociliz	tocilizumab	No steroids	1	90	127	33	NA
1, Gordon_	AP-CAP_1_NCT02735707_tociliz	standard care	No steroids	1	90	129	44	NA
1, Gordon_	AP-CAP_1_NCT02735707_sarilu	sarilumab	No steroids	1	90	4	2	NA
1, Gordon_	AP-CAP_1_NCT02735707_sarilu	standard care	No steroids	1	90	11	5	NA
Gordon_2	REMAP-CAP_2_NCT02735707	tocilizumab	Steroids	1	NR	589	215	NA
Gordon_2	REMAP-CAP_2_NCT02735707	sarilumab	Steroids	1	NR	431	146	NA
CTA_NCT0	REMDACTA_NCT04409262	tocilizumab	Steroids	1	28	358	69	NA
CTA_NCT0	REMDACTA_NCT04409262	placebo	Steroids	1	28	181	39	NA
CTA_NCT0	REMDACTA_NCT04409262	tocilizumab	No steroids	1	28	72	9	NA
CTA_NCT0	REMDACTA_NCT04409262	placebo	No steroids	1	28	29	2	NA
27470	TOCIBRAS_NCT04403685	tocilizumab	Steroids	1	90	31	9	NA
27470	TOCIBRAS_NCT04403685	standard care	Steroids	1	90	34	5	NA
27470	TOCIBRAS_NCT04403685	tocilizumab	No steroids	1	90	34	8	NA
27470	TOCIBRAS_NCT04403685	standard care	No steroids	1	90	30	2	NA
-SARI-1_N	ORIMUNO-SARI-1_NCT0432407	sarilumab	Steroids	1	90	4	2	NA
-SARI-1_N	ORIMUNO-SARI-1_NCT0432407	standard care	Steroids	1	90	3	0	NA
-SARI-1_N	ORIMUNO-SARI-1_NCT0432407	sarilumab	No steroids	1	90	72	14	NA
-SARI-1_N	ORIMUNO-SARI-1_NCT0432407	standard care	No steroids	1	90	65	10	NA
SARI-ICU_N	RIMUNO-SARI-ICU_NCT043240	sarilumab	Steroids	1	90	0	0	NA
SARI-ICU_N	RIMUNO-SARI-ICU_NCT043240	standard care	Steroids	1	90	2	0	NA
SARI-ICU_N	RIMUNO-SARI-ICU_NCT043240	sarilumab	No steroids	1	90	48	14	NA
SARI-ICU_N	RIMUNO-SARI-ICU_NCT043240	standard care	No steroids	1	90	31	13	NA
VID_NCT04	SARCOVID_NCT04357808	sarilumab	Steroids	1	90	17	1	NA
VID_NCT04	SARCOVID_NCT04357808	standard care	Steroids	1	90	8	0	NA
VID_NCT04	SARCOVID_NCT04357808	sarilumab	No steroids	1	90	3	1	NA
VID_NCT04	SARCOVID_NCT04357808	standard care	No steroids	1	90	2	0	NA
udraCT202	ARICOR_EudraCT2020-001531-2	sarilumab	Steroids	1	28	66	2	NA
udraCT202	ARICOR_EudraCT2020-001531-2	standard care	Steroids	1	28	34	3	NA
udraCT202	ARICOR_EudraCT2020-001531-2	sarilumab	No steroids	1	28	10	1	NA
udraCT202	ARICOR_EudraCT2020-001531-2	standard care	No steroids	1	28	5	1	NA
draCT2020	ARTRE_EudraCT2020-002037-1	sarilumab	Steroids	1	28	70	2	NA
draCT2020	ARTRE_EudraCT2020-002037-1	standard care	Steroids	1	28	70	1	NA
draCT2020	ARTRE_EudraCT2020-002037-1	sarilumab	No steroids	1	28	0	0	NA
draCT2020	ARTRE_EudraCT2020-002037-1	standard care	No steroids	1	28	0	0	NA
OVIDSTORI	COVIDSTORM	tocilizumab	Steroids	1	90	13	1	NA
OVIDSTORI	COVIDSTORM	standard care	Steroids	1	90	17	1	NA
OVIDSTORI	COVIDSTORM	tocilizumab	No steroids	1	90	0	0	NA
OVIDSTORI	COVIDSTORM	standard care	No steroids	1	90	9	0	NA
ImmCoVA	ImmCoVA	tocilizumab	Steroids	1	28	21	2	NA
ImmCoVA	ImmCoVA	standard care	Steroids	1	28	26	2	NA
ImmCoVA	ImmCoVA	tocilizumab	No steroids	1	28	1	0	NA
ImmCoVA	ImmCoVA	standard care	No steroids	1	28	1	0	NA
Rutgers	PreToVid_Trial NL8504	tocilizumab	Steroids	1	28	159	20	NA
Rutgers	PreToVid_Trial NL8504	standard care	Steroids	1	28	167	32	NA
Rutgers	PreToVid_Trial NL8504	tocilizumab	No steroids	1	28	11	1	NA
Rutgers	PreToVid_Trial NL8504	standard care	No steroids	1	28	11	2	NA
41237	Rashad	tocilizumab	NA	1	14	74	60	NA
41237	Rashad	examethasor	NA	1	14	75	45	NA
vapalasinga	REGENERON-P2_NCT04315298	sarilumab	Steroids	1	90	62	28	NA
vapalasinga	REGENERON-P2_NCT04315298	standard care	Steroids	1	90	14	6	NA
vapalasinga	REGENERON-P2_NCT04315298	sarilumab	No steroids	1	90	305	81	NA
vapalasinga	REGENERON-P2_NCT04315298	standard care	No steroids	1	90	76	18	NA
vapalasinga	REGENERON-P3_NCT04315298	sarilumab	Steroids	1	90	345	99	NA
vapalasinga	REGENERON-P3_NCT04315299	standard care	Steroids	1	90	99	34	NA
vapalasinga	REGENERON-P3_NCT04315298	sarilumab	No steroids	1	90	699	198	NA

ɪapalasinga	REGENERON-P3_NCT04315299	standard care	No steroids	1	90	187	50	NA
COVITOZ	COVITOZ	tocilizumab	Steroids	1	90	6	0	NA
COVITOZ	COVITOZ	standard care	Steroids	1	90	6	0	NA
COVITOZ	COVITOZ	tocilizumab	No steroids	1	90	4	0	NA
COVITOZ	COVITOZ	standard care	No steroids	1	90	3	0	NA
