# Supplementary material for: Use of tocilizumab and sarilumab alone or in combination with corticosteroids for covid-19: systematic review and network meta-analysis
Source: BMJ Med. 2022 Feb 28;1(1):e000036. doi: 10.1136/bmjmed-2021-000036 (PMC9978750; doi:10.1136/bmjmed-2021-000036)
Supplement: Supplementary data [file bmjmed-2021-000036supp002.pdf]

Comparison		Direct estimate			Indirect estimate			Network estimate								
		Relative estimate			Relative estimate			Relative estimate			Absolute estimate (per 1,000)					Reasons
Treatment 1	Treatment 2	oint estimat	CI lower limi	CI upper limi	oint estimat	CI lower limi	CI upper limi	Point estim	CI lower lin	CI upper lin	Point estim	CI lower lin	CI upper lin	Final rating	Higher certainty estimate	
sarilumab	sarilumab, steroids	NA	NA	NA	1.75	1.21	2.56	1.75	1.21	2.56	82.6	31.26	125	LOW	NMA	RoB, Imprecision
sarilumab	standard care/placebo	1.05	0.68	1.59	NA	NA	NA	1.07	0.81	1.4	10.6	-38.37	55.17	LOW	NMA	RoB, Imprecision
sarilumab	steroids	NA	NA	NA	1.28	0.95	1.72	1.28	0.95	1.72	39.87	-7.76	82.29	LOW	NMA	RoB, Imprecision
sarilumab	tocilizumab	NA	NA	NA	0.95	0.68	1.35	0.95	0.68	1.35	-9.13	-74.66	49.13	VERY LOW	NMA	RoB, Imprecisionx2
sarilumab	tocilizumab, steroids	NA	NA	NA	1.63	1.2	2.25	1.63	1.2	2.25	74.41	29.82	112.66	LOW	NMA	RoB, Imprecision
sarilumab, steroids	standard care/placebo	NA	NA	NA	0.61	0.47	0.79	0.61	0.47	0.79	-72	-106.11	-37.34	MODERATE	NMA	RoB
sarilumab, steroids	steroids	0.82	0.49	1.41	0.69	0.52	0.92	0.73	0.58	0.92	-42.73	-72.61	-12	LOW	NMA	RoB, Imprecision
sarilumab, steroids	tocilizumab	NA	NA	NA	0.54	0.39	0.76	0.54	0.39	0.76	-91.73	-144.09	-42.48	MODERATE	NMA	RoB
tocilizumab, steroids	sarilumab, steroids	1.12	0.34	3.7	0.95	0.63	1.42	1.07	0.86	1.34	8.19	-20.49	34.96	LOW	NMA	RoB, Imprecision
steroids	standard care/placebo	0.84	0.64	1.07	0.36	0.16	0.77	0.84	0.75	0.93	-29.27	-46.74	-12.24	MODERATE	NMA	RoB
tocilizumab	standard care/placebo	0.98	0.7	1.36	2.47	1.17	5.4	1.12	0.91	1.38	19.73	-15.78	58.52	LOW	NMA	RoB, Imprecision
standard care/placebo	tocilizumab, steroids	NA	NA	NA	1.54	1.31	1.79	1.54	1.31	1.79	63.81	40.66	88.06	MODERATE	NMA	RoB
tocilizumab	steroids	2.86	0.75	10.95	1.23	0.97	1.57	1.34	1.07	1.68	49	10.17	91.51	MODERATE	NMA	RoB
tocilizumab, steroids	steroids	0.8	0.64	1.01	0.91	0.57	1.46	0.79	0.7	0.88	-34.54	-51.8	-18.23	MODERATE	NMA	RoB
tocilizumab	tocilizumab, steroids	NA	NA	NA	1.72	1.32	2.23	1.72	1.32	2.23	83.54	41.39	129.99	MODERATE	NMA	RoB
