# Supplementary material for: Use of tocilizumab and sarilumab alone or in combination with corticosteroids for covid-19: systematic review and network meta-analysis
Source: BMJ Med. 2022 Feb 28;1(1):e000036. doi: 10.1136/bmjmed-2021-000036 (PMC9978750; doi:10.1136/bmjmed-2021-000036)
Supplement: Supplementary data [file bmjmed-2021-000036supp003.pdf]

		NMA estimate, odds ratio			NMA estimate, risk difference			Indirect estimate, odds ratio			Direct estimate, odds ratio		
**Treatment 1**	**Treatment 2**	**OR**	**LCL**	**UCL**	**RD**	**LCL**	**UCL**	**OR**	**LCL**	**UCL**	**OR**	**LCL**	**UCL**
sarilumab	sarilumab, steroids	1.75	1.07	2.92	82.4	12.1	136.9						
sarilumab	standard care/placebo	1.06	0.76	1.49	9.1	-52.1	62.6				1.05	0.68	1.59
sarilumab	steroids	1.34	0.92	2.01	46.7	-14.6	100.4						
**sarilumab**	**tocilizumab**	**0.97**	**0.64**	**1.48**	-8.2	-88.4	61.3						
sarilumab	tocilizumab, steroids	1.67	1.09	2.59	76.2	15.0	126.7						
sarilumab, steroids	standard care/placebo	0.61	0.41	0.87	-73.3	-121.2	-22.5						
**sarilumab, steroids**	**steroids**	**0.76**	**0.56**	**1.06**	-35.7	-74.2	8.1	0.71	0.44	1.15	0.82	0.49	1.41
sarilumab, steroids	tocilizumab	0.55	0.36	0.85	-90.6	-158.0	-26.4						
**tocilizumab, steroids**	**sarilumab, steroids**	**1.05**	**0.77**	**1.43**	6.2	-36.0	43.4	0.96	0.59	1.57	1.12	0.34	3.70
steroids	standard care/placebo	0.79	0.64	0.95	-37.5	-70.3	-8.2	0.35	0.15	0.80	0.84	0.64	1.07
tocilizumab	standard care/placebo	1.09	0.85	1.40	17.3	-25.7	65.5	2.42	1.07	5.59	0.98	0.70	1.36
standard care/placebo	tocilizumab, steroids	1.57	1.20	2.08	67.1	29.0	106.9						
tocilizumab	steroids	1.39	1.03	1.90	54.9	5.9	111.4	1.23	0.89	1.70	2.86	0.75	10.95
**tocilizumab, steroids**	**steroids**	**0.80**	**0.67**	**0.99**	-29.6	-55.2	-2.2	0.92	0.50	1.74	0.80	0.64	1.01
tocilizumab	tocilizumab, steroids	1.72	1.21	2.46	84.4	29.5	146.1						

OR, odds ratio; LCL, 95% lower credible limit; UCL, 95% upper credible limit
